# PD-L1 Expression Induced by the 2009 Pandemic Influenza A(H1N1) Virus Impairs the Human T Cell Response

**DOI:** 10.1155/2013/989673

**Published:** 2013-09-26

**Authors:** Nuriban Valero-Pacheco, Lourdes Arriaga-Pizano, Eduardo Ferat-Osorio, Luz María Mora-Velandia, Rodolfo Pastelin-Palacios, Miguel Ángel Villasís-Keever, Celia Alpuche-Aranda, Luvia Enid Sánchez-Torres, Armando Isibasi, Laura Bonifaz, Constantino López-Macías

**Affiliations:** ^1^Medical Research Unit on Immunochemistry (UIMIQ), Specialties Hospital, National Medical Centre “Siglo XXI,” Mexican Social Security Institute (IMSS), 06720 México, DF, Mexico; ^2^Departamento de Inmunología, Escuela Nacional de Ciencias Biológicas, Instituto Politécnico Nacional, 11340 México, DF, Mexico; ^3^Facultad de Química, Universidad Nacional Autónoma de México, 04510 México, DF, Mexico; ^4^Unidad de Investigación en Epidemiología Clínica, Hospital de Pediatría, Centro Médico Nacional Siglo XXI, Instituto Mexicano del Seguro Social (IMSS), 06720 México, DF, Mexico; ^5^Instituto Nacional de Salud Pública, 62100 Cuernavaca, MOR, Mexico

## Abstract

PD-L1 expression plays a critical role in the impairment of T cell responses during chronic infections; however, the expression of PD-L1 on T cells during acute viral infections, particularly during the pandemic influenza virus (A(H1N1)pdm09), and its effects on the T cell response have not been widely explored. We found that A(H1N1)pdm09 virus induced PD-L1 expression on human dendritic cells (DCs) and T cells, as well as PD-1 expression on T cells. PD-L1 expression impaired the T cell response against A(H1N1)pdm09 by promoting CD8^+^ T cell death and reducing cytokine production. Furthermore, we found increased PD-L1 expression on DCs and T cells from influenza-infected patients from the first and second 2009 pandemic waves in Mexico City. PD-L1 expression on CD8^+^ T cells correlated inversely with T cell proportions in patients infected with A(H1N1)pdm09. Therefore, PD-L1 expression on DCs and T cells could be associated with an impaired T cell response during acute infection with A(H1N1)pdm09 virus.

## 1. Introduction

Programmed death-ligand 1 (PD-L1, B7-H1, CD274) is a coinhibitory molecule that has been associated with impairment of the T cell response. PD-L1 is one of the ligands that interact with the inhibitory PD-1 receptor, which is expressed on activated T cells [1]. PD-L1 expression is induced in a variety of human cells and tissues, including T cells and dendritic cells (DCs) [2]. PD-1/PD-L1 signaling interferes with the T cell response by blocking the CD28-mediated pathway, thereby affecting the expression of antiapoptotic genes, cell cycle progression [3], and cytokine production [4]. The role of the PD-1/PD-L1 signaling pathway in chronic infections, such as HIV or HCV infection, has been widely explored [5]. PD-L1 signaling is involved in the induction of T cell exhaustion, which impairs the response against pathogens. Additionally, this pathway is important in regulating the balance between an effective antimicrobial response and tissue damage [5]. The role of PD-1/PD-L1 during acute infections has been studied in mouse models of rabies [6], influenza [7], sepsis [8], RSV, and HMPV, and in patients with septic shock [9] with divergent findings, most of which suggest an inhibitory role for PD-L1. Recently, the expression of PD-1 and PD-L1 in the lungs of patients infected with the 2009 pandemic influenza A(H1N1) virus (A(H1N1)pdm09) was documented [10]. During chronic viral infections, PD-L1 expression on T cells has been reported to be crucial in the impairment of the T cell response [5, 11]. However, PD-L1 expression on DCs and T cells during acute viral infections, particularly during A(H1N1)pdm09 infection, has not been widely studied.

Influenza virus infection may trigger an exacerbated immune response, which has been correlated with illness severity and sometimes death [12–14]. Lymphopenia is a clinical feature of influenza infections caused by seasonal influenza [15], avian H5N1 [16], and A(H1N1)pdm09 viruses [17]. With regard to the cellular immune response, leukocytes exposed to seasonal influenza virus have been shown to proliferate in response to the virus, but did not show a subsequent response to mitogen stimulation [18]. Additionally, influenza virus can induce apoptosis of several cell types, including peripheral blood-derived macrophages [19], avian cell lines [20], and T cells from healthy subjects [21].

Cellular immunity, may contribute to virus clearance, reduction of symptoms and prevention of secondary infections [22, 23]. The CD4^+^ T cell-mediated immune response against influenza plays a role in limiting the severity of infection in the absence of previous antibodies [24]. However, during the acute phase of infection, T cells from patients infected with A(H1N1)pdm09 cannot differentiate into effector cells, highly express the death receptor CD95 (Fas), and do not respond to mitogens; nevertheless, T cell function is restored during the convalescent phase [25]. Therefore, the lymphopenia and T cell dysfunction reported in the A(H1N1)pdm09 infection might be induced by PD-L1 expressed on T cells, which could have affected T cell function through a mechanism similar to that which has been reported in chronic viral infections. This study evaluated the expression of PD-L1 on DCs and T cells and its effects on T cell response, as well as its possible implications during A(H1N1)pdm09 infection at the beginning of the 2009 pandemic outbreak at its epicenter.

## 2. Materials and Methods

### 2.1. Patients and Healthy Controls

Thirteen patients from two hospitals from the Mexican Social Security Institute (IMSS) with RT-PCR-confirmed pandemic influenza infection (pH1N1+), 12 PCR negative patients with influenza-like illness (ILI) (pH1N1−), and 10 healthy controls (HC) were included in this report. Patients were recruited during the first and second pandemic waves in Mexico City. Informed consent was obtained from participants. Study approval was obtained from the IMSS through the National Commission of Scientific Research, which comprises the Scientific, Ethics, and Biosafety Committees, in accordance with Good Clinical Practice. The project's ethics authorization number is: CNIC 2010-785-002.

### 2.2. Blood Samples and PBMC Separation

Blood samples from patients and controls were collected in EDTA tubes. Peripheral blood mononuclear cells (PBMCs) were separated by gradient centrifugation using Lymphoprep (Axis-Shield, Oslo, Norway) and cryopreserved until use. PBMCs from buffy coats were obtained from healthy volunteer donors according to institutional guidelines.

### 2.3. PBMC Stimulation

PBMCs (1 × 10^6^) from buffy coats were placed in 24-well plates (Corning Inc., Corning, NY, USA) with RPMI-1640 (supplemented with HEPES, 10% heat-inactivated fetal bovine serum, 2 mM L-glutamine, 100 UmL^−1^ penicillin, and 100 *μ*gmL^−1^ streptomycin, all from Gibco, Life Technologies, Carlsbad, CA, USA). They were stimulated with 10 pgmL^−1^ staphylococcal enterotoxin B (SEB, Toxin Technology, Sarasota, FL, USA), 10 *µ*gmL^−1^ of the TLR7 synthetic agonist CL264 (Invivogen, San Diego, CA, USA), 80 HAUmL^−1^ (hemagglutination units) of live and UV-inactivated influenza A/Mexico/4482/2009(H1N1) virus and A/Panama/2007/1999(H3N2) virus provided by the Instituto Nacional de Referencia Epidemiológica (INDRE), or 10 *μ*gmL^−1^of recombinant A(H1N1)pdm09 virus hemagglutinin (HA), kindly provided by Dr. Clara Espitia from the Instituto de Investigaciones Biomédicas, UNAM. The PBMCs were incubated for 18 h, 3 or 7 days at 37°C/5% CO_2_ prior to flow cytometry analysis of PD-1 and PD-L1 expression on DCs and T cells, respectively. For *de novo* protein synthesis analysis, PBMCs were stimulated with A(H1N1)pdm09 for 2 h, then cycloheximide (CHX, 50 *μ*gmL^−1^) was added to the culture for another 16 h.

### 2.4. T Cell and Dendritic Cell Enrichment and Culture

PBMCs (2 × 10^7^) from buffy coats were incubated in supplemented RPMI-1640 at 37°C/5% CO_2_ for 1.5 h, in Petri dishes (Fisher Scientific, Pittsburgh, PA, USA). Nonadherent cells were removed, washed, and quantified. T cells were then isolated by negative selection using a cocktail of PE-conjugated anti-CD19, anti-CD14 (eBioscience, San Diego, CA), anti-CD56, and anti-HLA-DR antibodies (BD Biosciences, San Jose, CA), with anti-PE magnetic microbeads in a MidiMACS system with LD columns (Miltenyi Biotec, Auburn, CA, USA). Dendritic cells were isolated the same way, but instead of anti-HLA-DR, PE-conjugated anti-CD3 (eBioscience) was used.

### 2.5. Stimulation of Enriched T Cells and DCs

The enriched T cells (5 × 10^5^ cells/well) were placed into 48-well plates (Corning) with supplemented RPMI-1640 and stimulated with 10 pgmL^−1^ SEB and 80 HAU/mL A(H1N1)pdm09 virus and incubated for 48 h at 37°C/5% CO_2_. Enriched DCs (1.5 × 10^6^) were placed in 24-well plates (Corning) with supplemented RPMI-1640 and stimulated with CL264 or A(H1N1)pdm09 virus. The cells were incubated for 18 h at 37°C/5% CO_2_, collected, and labeled for flow cytometric analysis.

### 2.6. T Cell Proliferation and Cell Death

Buffy coat PBMCs (5 × 10^6^ cells/well) were left untreated or stimulated with influenza virus and incubated for 18 h. Next, the cells were labeled with CFSE (Invitrogen Life Technologies, Carlsbad, CA, USA), and 5 × 10^5^ cells/well were seeded into plates. These cells were left untreated or treated with 25 *μ*gmL^−1^ anti-PD-L1 antibody 29E.2A3 (BioLegend, San Diego, CA, USA) or an isotype control MPC-11 (BioLegend) on days 0, 3, and 5, or with SEB (10 pgmL^−1^) on day 0. The cells were incubated for 7 days at 37°C/5% CO_2_, collected, and proliferation was measured by flow cytometry. The proportion of apoptotic cells was detected by flow cytometry with Annexin V/Pacific Blue and 7-AAD staining (both from BioLegend).

### 2.7. Cytokine Production

Supernatants from the T cell proliferation culture were cryopreserved until use. Cytokine levels were measured using the human Th1/Th2/Th17 cytometric bead array kit (CBA) according to the manufacturer's instructions (BD Biosciences).

### 2.8. Sorting of cDCs and Isolation of CD4^+^ Memory T Cells

Sorting of cDCs from enriched DCs was performed in a FACSAria cell sorter (BD Biosciences). After the preenrichment previously described, the negative cell fraction was labeled with anti-CD123-/PE-Cy5 and anti-HLA-DR/APC-Cy7 (BioLegend), to identify the cDC population; this population was isolated with a purity of about 90%. Memory CD4^+^ T cells (T_m_) were isolated with the human memory CD4^+^ T cell isolation kit (Miltenyi Biotec). For co-culture assays, a 1 : 3 ratio of cDCs : T cells (1.5 × 10^4^ cDCs and 4.5 × 10^4^ T cells) were placed in 96-well plates with A(H1N1)pdm09 virus and incubated for 7 days at 37°C/5% CO_2_, with or without PD-L1 blocking; the supernatant was collected to conduct CBA's analysis. Representative plots of cDCs and CD4^+^ T cell purity are shown in Figure S1 (see Supplementary Material available online at http://dx.doi.org/10.1155/2013/989673).

### 2.9. Flow Cytometric Analysis

The cryopreserved PBMCs from patients and controls were thawed and counted. Only the samples with total PBMCs above 1 × 10^6^ cells were evaluated for PD-L1 expression on both DCs and T cells. When the number of cells was inferior, only DCs or T cells were analyzed. Hence, PD-L1 expression was analyzed on T cells of 9/13 pH1N1+ patients and 6/12 pH1N1− patients. In the case of DCs, PD-L1 expression was evaluated in 11/13 pH1N1+ patients and 10/12 in the pH1N1− group. The PBMCs from patients were labeled with the fluorochrome-conjugated antibodies, PD-L1/PE-Cy7, CD8/APC-Cy7, HLA-DR/APC-H7, CD4/PE-Cy7, CD123/PE-Cy5, and the lineage cocktail (Lin, CD3/PE, CD14/PE, CD56/PE, and CD19/PE) (BD Biosciences) and fixed with paraformaldehyde (4%). The T cells and DCs from buffy coats used for *in vitro* PD-L1 expression, proliferation, and cell death assays were labeled with all of the former antibodies, in addition to PD-1/FITC, CD4/APC-Cy7, CD2/PE, (BD Biosciences), and CD8/APC (Invitrogen). The cells were fixed with FACS Lysing Solution 1x (BD Biosciences) and cell viability was determined with Hoechst 33258 staining (Invitrogen). All samples were analyzed in either a FACSAria II or a FACSCanto II (BD Biosciences) using FlowJo (version 8.7) software (Tree Star Inc., Ashland, OR, USA). A minimum of 1 × 10^4^ CD2^+^ events were collected for T cell samples and 5 × 10^4^ Lin-events for DCs samples.

### 2.10. Statistical Analysis

Statistics were calculated with Prism, version 5.0, from GraphPad Software (San Diego, CA, USA). To test for significant differences in PD-L1 expression between treatments, MFI values were normalized and a parametric Student's *t*-test with a two-tailed *P* value was performed. In the case of patients, a nonparametric Student's *t*-test was performed (Mann-Whitney test). Correlations were established with Spearman's test. Statistical significance was established at *P* < 0.05.

## 3. Results

### 3.1. PD-L1 Is Expressed on Human Dendritic Cells and T Cells, Whereas PD-1 Is Only Expressed on T Cells after Exposure to A(H1N1)pdm09 Virus

**Figure 1:**
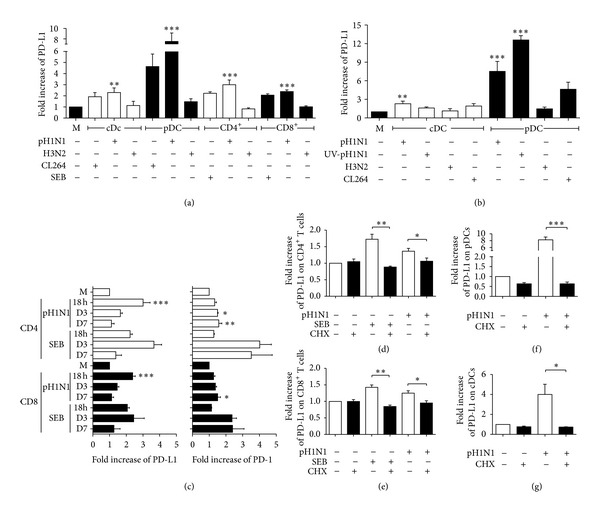
PD-L1 is expressed on human dendritic cells and T cells, whereas PD-1 is expressed only on T cells after exposure to A(H1N1)pdm09 virus. PBMCs were stimulated with A(H1N1)pdm09 virus (pH1N1), seasonal influenza virus (H3N2), staphylococcal enterotoxin B (SEB), or synthetic TLR7 agonist (CL264); PD-L1 and PD-1 expression on DCs and T cells was analyzed by flow cytometry. (a) Fold increase in PD-L1 expression on conventional (cDCs) and plasmacytoid dendritic cells (pDCs), CD4^+^, and CD8^+^ T cells after 18 h of stimulus. M: medium. (b) PBMCs were stimulated with live or UV-inactivated pH1N1 for 18 h; virus and PD-L1 expression was measured on cDCs and pDCs. (c) Kinetics of PD-L1 and PD-1 expression on CD4^+^ and CD8^+^ T cells induced by pH1N1 or SEB. PBMCs were stimulated with pH1N1 for 2 h, then cycloheximide (CHX) was added for another 16 h, and PD-L1 expression on CD4^+^(d), CD8^+^ T cells (e), pDCs (f), and cDCs (g) was measured by flow cytometry. (*n* = 5 donors, error bars indicate standard error of the mean (SEM)). *P < 0.05, **P < 0.01, and ***P < 0.001 by one way ANOVA test with Bonferroni posttest.

To test whether A(H1N1)pdm09 could induce PD-L1 and PD-1 expression on DCs and T cells, we stimulated human PBMCs with A(H1N1)pdm09. After 18 h of contact with the virus, we detected PD-L1 expression on conventional (cDCs) (*P* < 0.01) and plasmacytoid dendritic cells (pDCs) (*P* < 0.001) (Figure 1(a)). This was similar to the expression induced by the synthetic TLR7 agonist CL264. The A(H1N1)pdm09 virus induced PD-L1 on both CD4^+^ (*P* < 0.001) and CD8^+^ T cells (*P* < 0.001) similar to SEB (Figure 1(a)). We did not observe H3N2 seasonal virus induction of PD-L1 expression on any of the analyzed cells. In addition, after stimulation for 18 h with A(H1N1)pdm09 virus, we detected no PD-1 expression on DCs or T cells (Figure S2(a)). To evaluate whether PD-L1 expression on DCs could be related to viral infection, we stimulated DCs with live or UV-inactivated A(H1N1)pdm09 virus; we did not detect any significant differences in PD-L1 expression induced by live or UV-inactivated virus on DCs populations (Figure 1(b)), although it was slightly decreased in cDCs treated with the inactivated virus. Next, we considered the possibility that the kinetics of PD-1 and PD-L1 expression on T cells could be divergent; therefore, we analyzed the expression of these molecules over 7 days after A(H1N1)pdm09 stimulation; the highest expression of PD-L1 on both CD4^+^ and CD8^+^ T cells was detected after 18 h and decreased over time (*P* < 0.001) (Figure 1(c)). In the case of PD-1, we only observed significant differences after 3 days of virus stimulation in CD4^+^ T cells and after 7 days in CD8^+^ T cells (*P* < 0.05) (Figure 1(c)). To elucidate whether PD-L1 expression on DCs and T cells could be caused directly by interaction with the virus, we stimulated enriched DCs and HLA-DR^+^ cell-depleted T cells with A(H1N1)pdm09. We found that PD-L1 expression on DCs was induced after interaction with the virus and was dependent on *de novo* protein synthesis (Figures 1(f) and 1(g)); however, PD-L1 expression induced on T cells by A(H1N1)pdm09 was dependent on the presence of APCs in the culture (Figure S2(b)), and on *de novo* protein synthesis (Figures 1(d) and 1(e)). When we stimulated PBMCs with A(H1N1)pdm09 virus for 2 h, and then added cycloheximide for 16 h, PD-L1 expression on both CD4^+^ and CD8^+^ T cells was inhibited. (Figures 1(d) and 1(e)). These results indicate that A(H1N1)pdm09 can induce PD-L1 expression directly on human DCs and in the case of T cells by *de novo* protein synthesis, albeit dependent on the presence of APCs as an early event. PD-1 expression on DCs was absent, and in the case of T cells, it was induced by A(H1N1)pdm09 later in time.

### 3.2. PD-L1 Signaling Impairs T Cell Response against Pandemic A(H1N1)pdm09 Virus

**Figure 2:**
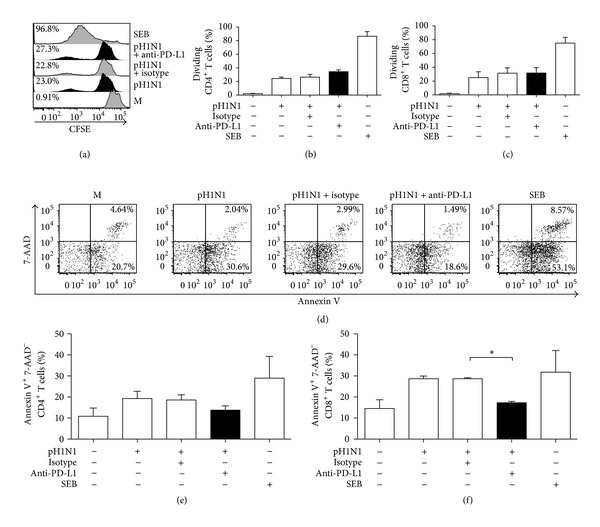
PD-L1 signaling blockade decreased CD8^+^ T cell death *in vitro* but did not have an effect on T cell proliferation in response to A(H1N1)pdm09 virus. PBMCs from healthy individuals were stimulated with A(H1N1)pdm09 for 18 h, washed, labeled with CFSE, and treated on days 0, 3, and 5 with a blocking anti-PD-L1 antibody or an isotype control. Cells were incubated for 7 days, and T cell proliferation and cell death were determined. SEB was used as a control. (a) Representative histograms of the CD4^+^ T cell CFSE dilution from one individual. (b, c) T cell proliferation expressed as the percentage of CFSE^+^ dividing cells. (d) Representative plot of Annexin V and 7-AAD staining to evaluate CD8^+^ T cell apoptosis, which was gated from CD2^+^ and CD8^+^ cells. (e, f) Percentage of early apoptotic (Annexin V^+^ 7-AAD^−^) T cells. (*n* = 7, error bars indicate SEM). *P < 0.05 by Student's *t*-test.

**Figure 3:**
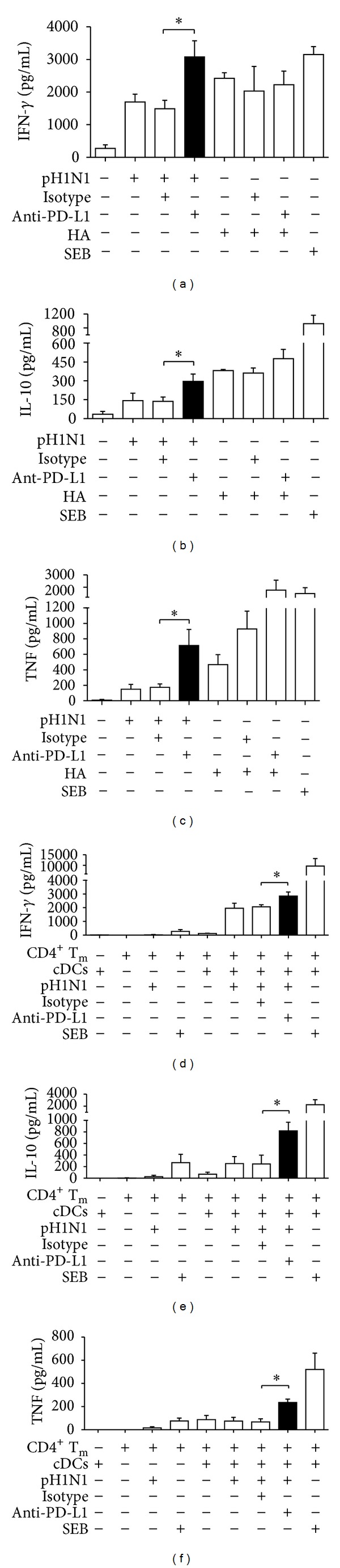
PD-L1 blocking increased *in vitro* IFN-*γ*, IL-10, and TNF production, predominantly by CD4^+^ T cells in response to A(H1N1)pdm09 virus. Cytokine levels in the supernatants (SN) of PBMCs cultured for 7 days as described in Figure 2 and PBMCs stimulated with hemagglutinin (HA) for 7 days were measured with a Th1/Th2/Th17 human cytometric bead array kit (CBA). The production of IFN-*γ* (a), IL-10 (b), and TNF (c) by PBMCs is shown. Isolated memory CD4^+^ T cells (T_m_) and sorted cDCs were co-cultured with or without PD-L1 blocking for 7 days, and cytokine production in the SNs was measured; IFN-*γ* (d), IL-10 (e), and TNF (f) levels are shown. Results are duplicates from 3 independent experiments and error bars indicate SEM. pH1N1: A(H1N1)pdm09 virus; SEB: staphylococcal enterotoxin B. *P < 0.05 by Student's *t*-test.

We analyzed if PD-L1 expression induced by A(H1N1)pdm09 could impair the T cell response against the virus. We blocked PD-L1 signaling during virus-induced T cell activation and established that blocking PD-L1 did not compromise T cell proliferation induced by the A(H1N1)pdm09 virus (Figures 2(a)–2(c)). However, we did observe that blocking PD-L1 decreased the proportion of early apoptotic (Annexin V^+^ 7-AAD^−^) CD8^+^ T cells, 7 days after exposure to the H1N1 virus (Figures 2(d) and 2(f), *P* < 0.05). CD4^+^ T cells did not show any significant differences in apoptosis after PD-L1 blocking (Figure 2(e)). We also determined that blocking PD-L1 increased PBMCs' production of IFN-*γ*, IL-10, and TNF. In order to evaluate if the virus caused this blocking effect, we stimulated the PBMCs with the recombinant hemagglutinin (HA) of the A(H1N1)pdm09 virus and measured cytokine production. We observed lower levels of IFN-*γ*, IL-10, and TNF when the whole A(H1N1)pdm09 virus was added, compared to HA stimulation. Moreover, when PD-L1 signaling was blocked, the cytokine levels induced by the virus were higher than those induced by the hemagglutinin. HA cytokine production was not affected by PD-L1 blockade, suggesting that this effect is A(H1N1)pdm09 virus-dependent (Figures 3(a)–3(c), *P* < 0.05). To evaluate if IFN-*γ*, IL-10, and TNF were mainly expressed by CD4^+^ T cells, we co-cultured isolated memory CD4^+^ T cells with sorted cDCs with or without PD-L1 blocking, and found that cytokine production by CD4^+^ T cells was dependent on the presence of cDCs and increased when PD-L1 was blocked (Figures 3(d)–3(f), *P* < 0.05). Neither in bulk PBMCs nor in co-cultures of purified cells did we observe an effect of PD-L1 blocking on IL-4, IL-17A, or IL-6 production (data not shown). In addition, we found that after 7 days of culture, enriched cDCs still expressed PD-L1 after stimulation with A(H1N1)pdm09 virus, in contrast, high expression of PD-L1 was observed in memory CD4^+^ T cells even in the absence of virus stimulation (Figure S3). Together, these results indicate that blocking PD-L1 on PBMCs had no effect on T cell proliferation but significantly decreased CD8^+^ T cell apoptosis and increased IFN-*γ*, IL-10, and TNF production by CD4^+^ T cells.

### 3.3. PD-L1 Expression Is Increased on Dendritic Cells and T Cells from PBMCs of Patients Infected with A(H1N1)pdm09 Virus

We evaluated PD-L1 expression in A(H1N1)pdm09 infection. We analyzed PD-L1 expression on T cells and DCs from cryopreserved PBMCs collected from patients during the 2009 influenza pandemic. Our study included 25 patients with influenza-like illness and 10 HCs, as shown in Table 1. Thirteen patients were RT-PCR positive for infection with the A(H1N1)pdm09 virus (pH1N1+), and the rest were categorized as pH1N1−. The median age of the patients was 48.5 years for H1N1− and 25 for H1N1+. Lymphopenia was present in most of the pH1N1+ patients (1403.8 ± 695.84 cells/mm^3^) and in half of the pH1N1− patients (1913.0 ± 1243.49 cells/mm^3^). We evaluated the frequencies and phenotype of DCs and T cells by flow cytometry; the gating strategy and representative plots are shown in Figure S4.

**Figure 4:**
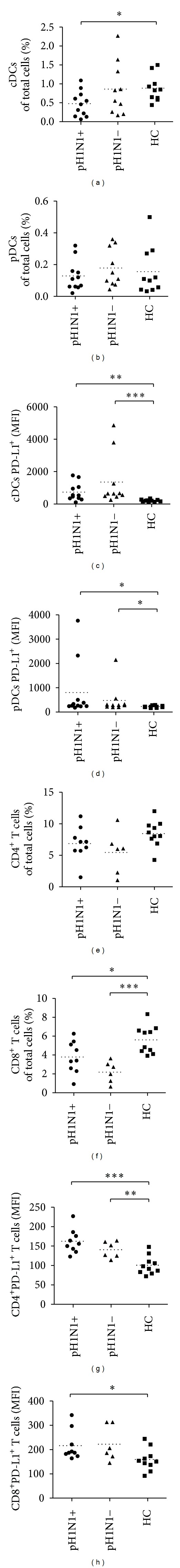
PD-L1 expression is increased on dendritic cells and T cells from PBMCs of patients with acute influenza infection. Cell proportions and surface PD-L1 expression on cDCs and pDCs (a–d) and CD4^+^ and CD8^+^ T cells (e–h) from cryopreserved PBMCs from patients with confirmed infection with A(H1N1)pdm09 virus (pH1N1+), patients with influenza-like illness but with a negative RT-PCR result for pandemic H1N1 influenza (pH1N1−), and healthy controls (HC, *n* = 10; error bars indicate SEM) were analyzed by flow cytometry. MFI: mean fluorescence intensity; pH1N1: A(H1N1)pdm09 virus. *P < 0.05, **P < 0.01, and ***P < 0.001 (Mann-Whitney test).

We found that pH1N1+ patients had a lower proportion of cDCs compared to the HCs (Figure 4(a), *P* < 0.05). No differences in the pDCs proportions between both groups were detected (Figure 4(b)). However, PD-L1 expression was increased on the cDCs and pDCs of both groups of patients compared to that of the HCs (Figure 4(c) pH1N1+, *P* < 0.01; pH1N1−, *P* < 0.001, and Figure 4(d), *P* < 0.05). CD4^+^ T cells proportion tended to decrease in both groups of patients compared to that of HCs (Figure 4(e)). The CD8^+^ T cell proportion was decreased in both groups of patients when compared to HCs (Figure 4(f), pH1N1+ *P* < 0.05, pH1N1−  *P* < 0.001). Relative PD-L1 expression was increased on CD4^+^ T cells in both groups of patients compared to that in HCs (Figure 4(g), pH1N1+  *P* < 0.001, pH1N1−  *P* < 0.01), while in CD8^+^ T cells, it was only increased in pH1N1+ patients (Figure 4(h), *P* < 0.05).

### 3.4. PD-L1 Expression on CD8^+^ T Cells Is Associated with a Lower T Cell Proportion in Patients Infected with A(H1N1)pdm09 Virus

**Figure 5:**
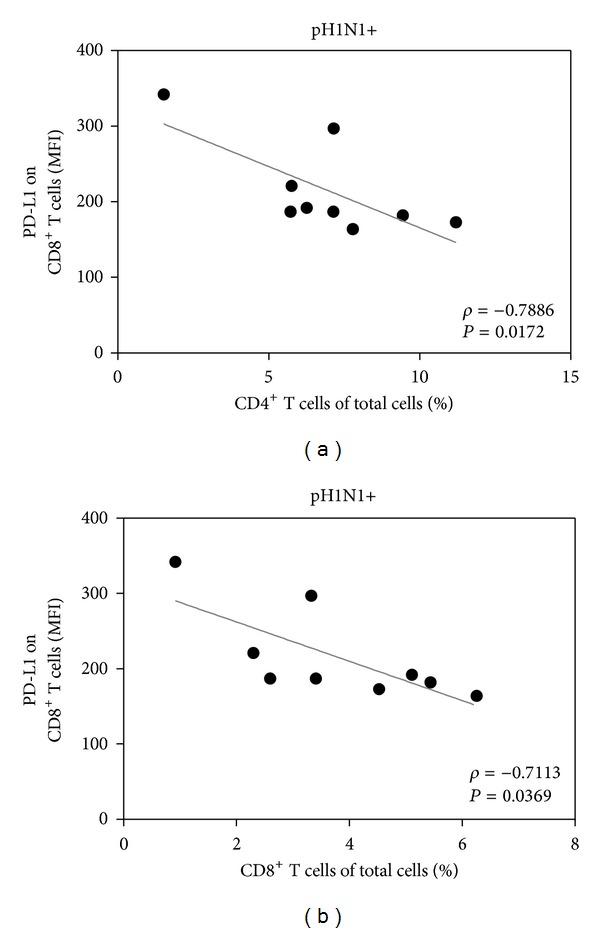
PD-L1 expression on CD8^+^ T cells is associated with a decreased T cell proportion in patients with acute A(H1N1)pdm09 viral infection. Correlations between PD-L1 expression on the proportions of CD8^+^ and CD4^+^ T cells in PBMCs from pH1N1+ patients are shown. Correlations between PD-L1 expression on CD8^+^ T cells and the proportion of total CD4^+^ (a) and CD8^+^ (b) T cells in PBMCs in pH1N1+ patients are indicated. Spearman correlation (*ρ*) and *P* values are shown in each graph. MFI: mean fluorescence intensity; pH1N1: A(H1N1)pdm09 virus.

Finally, to establish if PD-L1 expression in PBMCs from patients could be associated with the T cell proportion during infection, we performed a series of correlations of DCs and T cell proportions and determined PD-L1 expression in pH1N1+ and pH1N1− subjects. We detected an inverse correlation between PD-L1 expression on CD8^+^ T cells and the proportion of both T cell subsets only in pH1N1+ patients (Figures 5(a) and 5(b), *P* < 0.05); we did not find a significant correlation between PD-L1 expression and cell proportion in pH1N1− subjects or in DCs subsets (data not shown). As a whole, these results suggest that PD-L1 expression on T cells could be one of the factors mediating the decrease in the T cell proportion in pHN1+ patients.

## 4. Discussion

PD-L1 expression plays a critical role in chronic infections by impairing T cell function [5]. We report here that PD-L1 expression on DCs and T cells impairs T cell response to the influenza A(H1N1)pdm09 virus *in vitro*. We also suggest that PD-L1 expression could have implications during the acute natural infection.

A(H1N1)pdm09 was able to induce PD-L1 expression on DCs in a similar manner to a TLR7 ligand. It has been documented that TLR7 and retinoid-induced gene receptor 1 (RIG-1) mediate the recognition of influenza virus in DCs [26]. Therefore, human peripheral DCs may recognize the A(H1N1)pdm09 influenza virus through these receptors and subsequently express PD-L1 through a mechanism similar to that reported in influenza and other viral infections [6, 27]. PD-1 and PD-L1 expression can be induced on T cells through TCR signaling [2, 28]. We found that the PD-L1 expression induced by A(H1N1)pdm09 on T cells was APC-dependent, and mostly hinged on *de novo* protein synthesis. Additionally, we show that H3N2 seasonal virus failed to induce PD-L1 expression on either DCs or T cells; considering UV-inactivated pH1N1 induced PD-L1, it is possible that the expression of PD-L1 observed is independent of the infection capacity of the viruses. According to these results, we conclude that *in vitro*, there is an important difference between the pandemic and the seasonal influenza viruses in terms of their ability to induce PD-L1 expression. Furthermore, we found that PD-1 up regulation was detected at late time points in CD4^+^ T and CD8^+^ T cells cultures, after contact with the A(H1N1)pdm09 virus. This result is concordant with those observed in a recent mouse infection model, in which cognate viral antigen was necessary and sufficient to induce PD-1 expression on T cells, and that PD-1 was expressed by lymphocytes in the lower airways during acute influenza infection in humans [10].

PD-L1 expression has been associated with T cell exhaustion and dysfunction during chronic viral infections and in some acute infections in both *in vitro* and *in vivo* models [6, 27]. We have shown that similar T cell impairment mechanisms might also develop after interaction with A(H1N1)pdm09. Blocking PD-1/PD-L1 interaction enhanced the T cell response against A(H1N1)pdm09 virus. Apoptosis was significantly decreased in CD8^+^ T cells, whereas cytokine production was increased; however, no impact was observed on T cell proliferation. One explanation for these results could be the late up regulation of PD-1 expression on T cells. Therefore, T cell proliferation may not be affected by the PD-L1 blockade because PD-1 expression was not apparent until day 3; however, the effects that we observed on CD4^+^ T cell differentiation (cytokine production) when PD-L1 signaling was blocked, could be attributed to their expression of PD-1 until day 3. In addition, PD-L1 expression is maintained over time (7 days) in A(H1N1)pdm09 stimulated cDCs and is highly expressed on memory CD4^+^ T cells, indicating that these cells could be a source of PD-L1 during the late phase of T cell differentiation. Moreover, PD-1 is expressed on CD8^+^ T cells 7 days after A(H1N1)pdm09 stimulation, which correlates with decreased T cell death. There are previous reports suggesting that after direct virus exposure, human CD8^+^ T cells are more susceptible to apoptosis than CD4^+^ T cells [21]. In agreement with this finding, we observed that blocking PD-L1 after stimulation with A(H1N1)pdm09 could prevent CD8^+^ but not CD4^+^ T cell death.

Interestingly, we also observed that blocking PD-L1 caused an increase in the production of IL-10, IFN-*γ*, and TNF that may be associated with impairment of T cell differentiation induced by the virus, because when we stimulated cells with HA and blocked the PD-1/PD-L1 interaction, we did not observe any effects on cytokine production. The A(H1N1)pdm09 virus has also been reported to induce a decrease in cytokine levels in human DCs when compared with seasonal viruses *in vitro* [29]. Our study shows that PD-L1 expression induced by A(H1N1)pdm09 could inhibit the production of both inflammatory and regulatory cytokines in human bulk PBMCs and in co-cultures of purified cDCs and memory CD4^+^ T cells. It has been established that T cells from patients infected with A(H1N1)pdm09 cannot differentiate into effector cells, do not respond to mitogens, and highly express CD95 (Fas), suggesting an apoptosis-related mechanism for the lymphopenia reported in A(H1N1)pdm09 infection [25]. Furthermore, these findings could contribute to understanding the regulation of cytokine expression and the control of the exacerbated immune response during infection, as has been previously reported [12].

We observed low frequency of cDCs in the blood of patients infected with A(H1N1)pdm09 during the first and second pandemic waves in Mexico City. It has been reported that in influenza-infected patients, DCs are recruited in the lung, suggesting that the low proportions that we observed may be caused by the redistribution of the DC population from the blood to the lung [30]. Moreover, we observed a decrease in the proportion of CD8^+^ T cells in influenza-infected patients; thus, CD8^+^ T cells may also have been redistributed to the lungs. However, we found an increase in PD-L1 expression in DCs and T cells of pH1N1+ patients; it is possible that PD-1/PD-L1 signaling enhanced CD8^+^ T cell apoptosis as reflected in the decreased T cell proportion and as we showed in the *in vitro* assays results. 

Our *in vitro* results showed that unlike the A(H1N1)pdm09 virus, the H3N2 virus did not induce PD-L1 expression either in DCs or T cells; however, in addition to the seasonal H3N2 virus, seasonal H1N1 viruses were also circulating at that time in Mexico, so we cannot rule out that seasonal H1N1 virus could have also induced PD-L1 expression. Considering that our *ex vivo* results showed that PD-L1 up regulation may not be strain specific, and that in the natural infection additional immune mediators may contribute to PD-L1 up regulation, we do not discard the possibility that different types of influenza A virus could induce PD-L1 expression on DCs and T cells during acute infection. 

PD-1 and PD-L1 have been recently reported to be expressed in the lungs of A(H1N1)pdm09 patients [10]. Since we did not analyze respiratory tissue samples, it was not possible to determine if the consequences of PD-L1 expression on T cells and DCs that we observed in peripheral blood could reflect the localized response in lungs.

In A(H1N1)pdm09 infected patients, PD-L1 expression on CD8^+^ T cells is inversely correlated with CD4^+^ and CD8^+^ T cell proportions, but this correlation was only observed in pH1N1+ patients; this finding could be explained by the fact that the lymphopenia induced by A(H1N1)pdm09 has been reported to be more severe and refractory than that associated with seasonal infection, which is modest during the first days and resolves earlier [31]. Since we detected PD-L1 expression in both pH1N1+ and pH1N1− patients, we consider that additional factors related to the immune response and inflammation triggered during the acute infection (such as interferons), could be involved in the correlation between PD-L1 expression on CD8^+^ T cells and the proportion of T cells observed only in pH1N1+ patients [32, 33].

Our data suggest that viral infection may impair the induction of an efficient adaptive immune response in the early stages of infection by promoting PD-L1 expression on DCs and T cells; this could be a mechanism of immune evasion by the A(H1N1)pdm09 virus, similar to that reported in chronic and acute viral infections [6, 27, 34–36]. Since the analyzed patients were recruited at the beginning of the pandemic outbreak in Mexico City, whether these observations are a particular characteristic of early pandemic outbreaks or can also be observed during seasonal outbreaks remains to be elucidated. Our findings suggest that PD-L1 expression could be a useful marker in the evaluation of the early T cell response against influenza infection and may be a possible target for intervention in patients with other acute viral respiratory infections.

## 5. Conclusion

The 2009 pandemic influenza A(H1N1) virus is able to impair T cell responses through PD-L1 expression, suggesting that the virus could modulate host immune responses during infection by this mechanism.

## Supplementary Material

Figure S1. Representative plots of sorted conventional dendritic cells (cDCs) and isolated memory CD4^+^ T cells (T_m_). (a) Gating strategy reflecting the purity of isolated memory CD4^+^T cells (T_m_) characterized by the CD3^+^CD4^+^CD45RO^+^CD45RA^−^ phenotype (purity of 99.6%). (b) Gating strategy for sorting cDCs. (c) Gating strategy to phenotype cDCs, purity of 90.0% of lineage negative cDC population (CD3^−^, CD14^−^, CD19^−^, CD56^−^), HLA-DR^+^, and CD123^dim^.
Figure S2. The A(H1N1)pdm09 virus does not induce PD-1 expression;whereas PD-L1 expression on DCs is induced directly by the A(H1N1)pdm09 virus whilst PD-L1 expression on T cells is dependent on the presence of antigen-presenting cells. (a) PBMCs were stimulated with A(H1N1)pdm09 virus (pH1N1), seasonal influenza virus (H3N2), staphylococcal enterotoxin B or synthetic TLR7 agonist (CL264); PD-1 expression in DCs and T cells was analyzed by flow cytometry. Fold increase in PD-1 expression in cDCs and pDCs, CD4^+^ and CD8^+^ T cells after 18 h of stimulus. Enriched (HLA-DR^+^ cell-depleted)T cells and DCs (b) were stimulated with pH1N1, SEB or CL264; PD-L1 expression in DCs and T cells was analyzed by flow cytometry and representative histograms are shown. M: medium.
Figure S3. PD-L1 is expressed in cDCs and memory CD4^+^ T cells after 5 and 7 days of culture with A(H1N1)pdm09. (a) PD-L1 expression on isolated memory CD4^+^ T cells, 7 days after co-culture with sorted cDCs in the presence (blue) or absence (red) of pH1N1 virus. (b) PD-L1 expression on cDCs cultured for 5 days in the presence (blue) or absence (red) of pH1N1.
Figure S4. Gating strategy and representative plots of analyzed dendritic (DCs) and T cells from patients and healthy controls. Gating strategy and representative histograms of PD-L1 expression in cDCs (a, Lin^−^HLA-DR^+^CD123^dim^) and pDCs (b, Lin^−^HLA-DR^+^CD123^+^). Gating strategy and representative histograms of PD-L1 expression in CD4^+^ T cells (c, CD4^+^CD8^−^) and CD8^+^ T cells (d, CD4^−^CD8^+^). The shaded histogram represents PD-L1 expression in a healthy control, whereas the blue and red histograms are representative of two pH1N1+patients.

## Figures and Tables

**Table 1:** Demographic data from patients.

Variable	Total patients (*n* = 25)	
	H1N1− (*n* = 12)	H1N1+ (*n* = 13)
Gender		
Female	5	5
Male	7	8
Age (years)		
Mean	46.8	34.4
Median	48.5	25.0
Max	76	78
Min	18	17
Leukocyte count (cells/mm^3^) (mean ± SD)	7095.0 ± 4178.43	7643.8 ± 5122.75
Lymphocyte count (cells/mm^3^) (mean ± SD)	1913.0 ± 1243.49	1403.8 ± 695.84

## Abbreviations

PD-L1: Programmed death ligand-1
PD-1: Programmed death-1
cDC: Conventional dendritic cell
pDC: Plasmacytoid dendritic cell
A(H1N1)pdm09/pH1N1: 2009 pandemic influenza A(H1N1) virus
HC: Healthy control
PBMC: Peripheral blood mononuclear cells
SEB: Staphylococcal enterotoxin B
HA: Hemagglutinin.
