# Supplementary figures and images for: PD-L1 Expression Induced by the 2009 Pandemic Influenza A(H1N1) Virus Impairs the Human T Cell Response
Source: Clin Dev Immunol. 2013 Sep 26;2013:989673. doi: 10.1155/2013/989673 (PMC3803123; doi:10.1155/2013/989673)

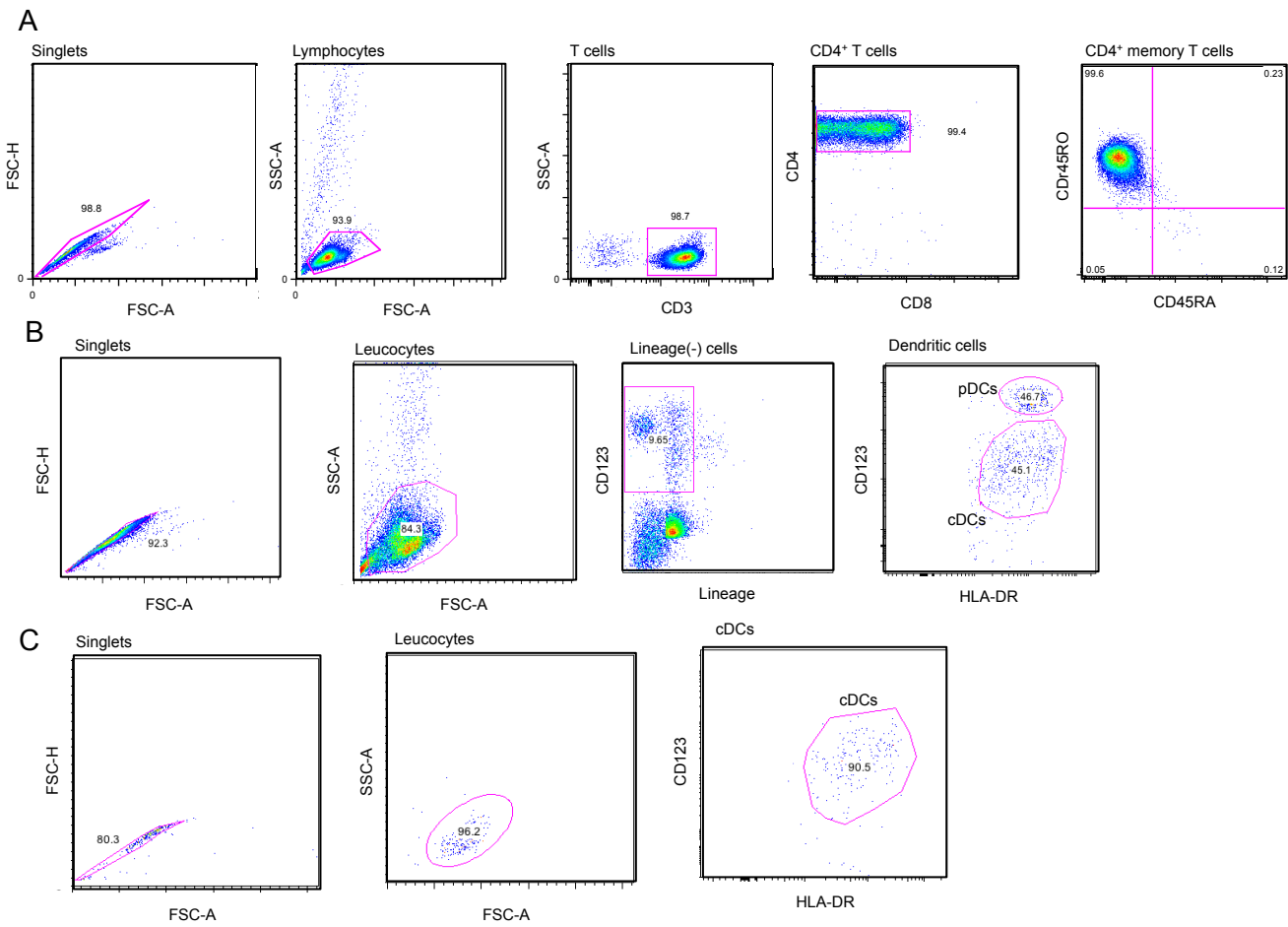

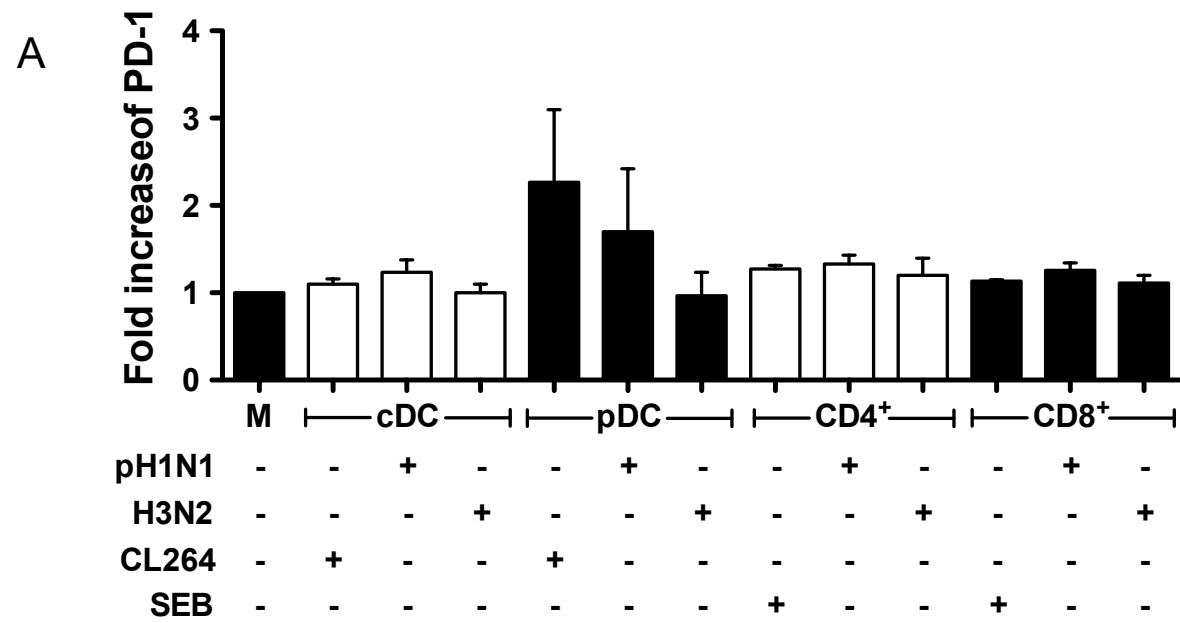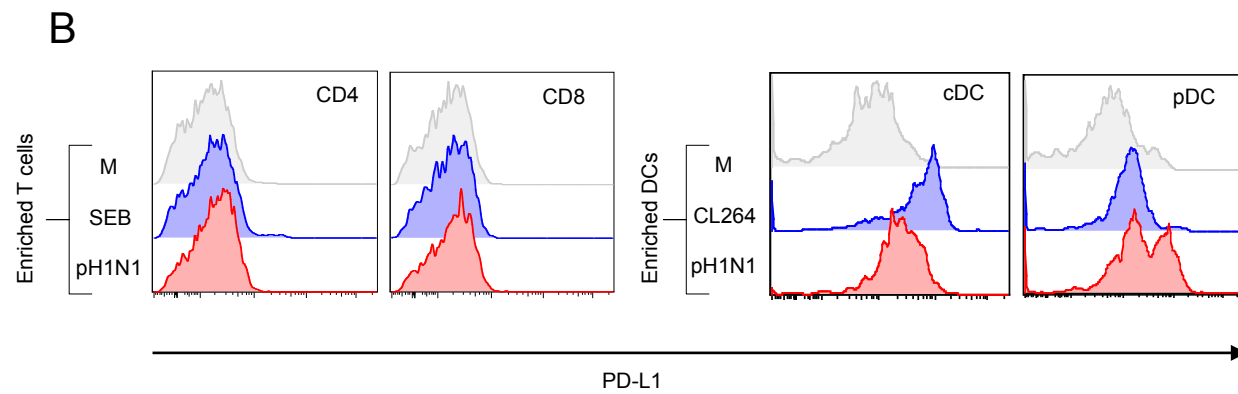

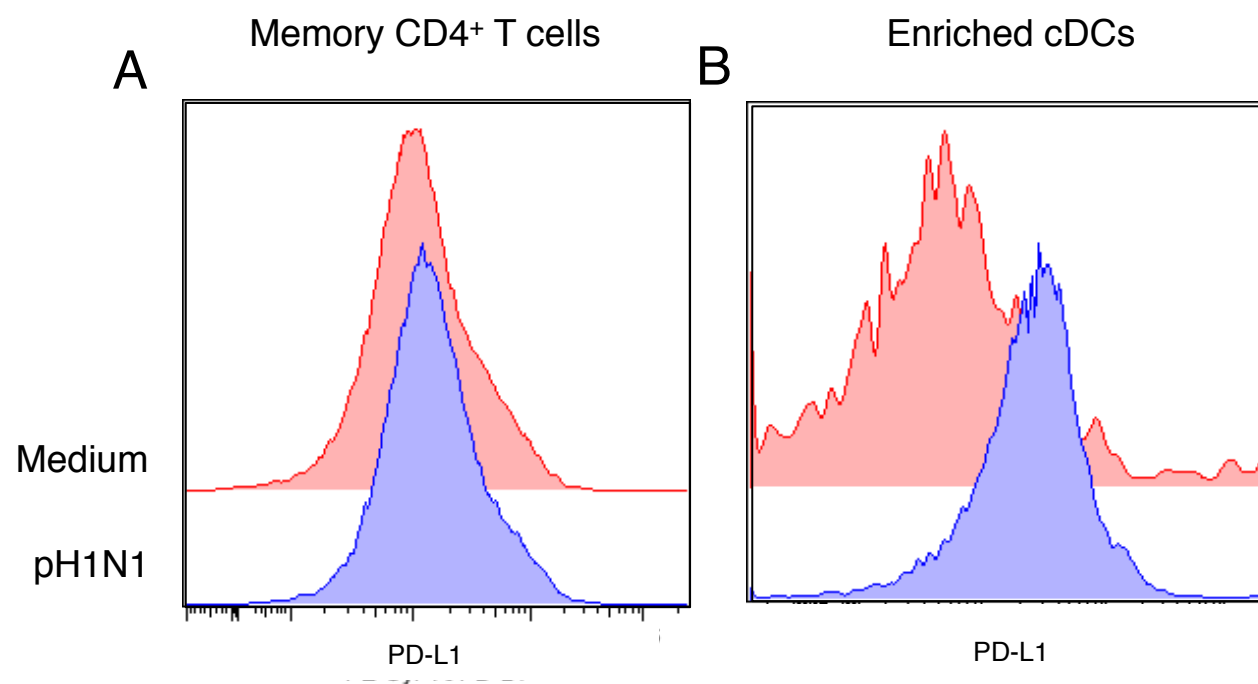

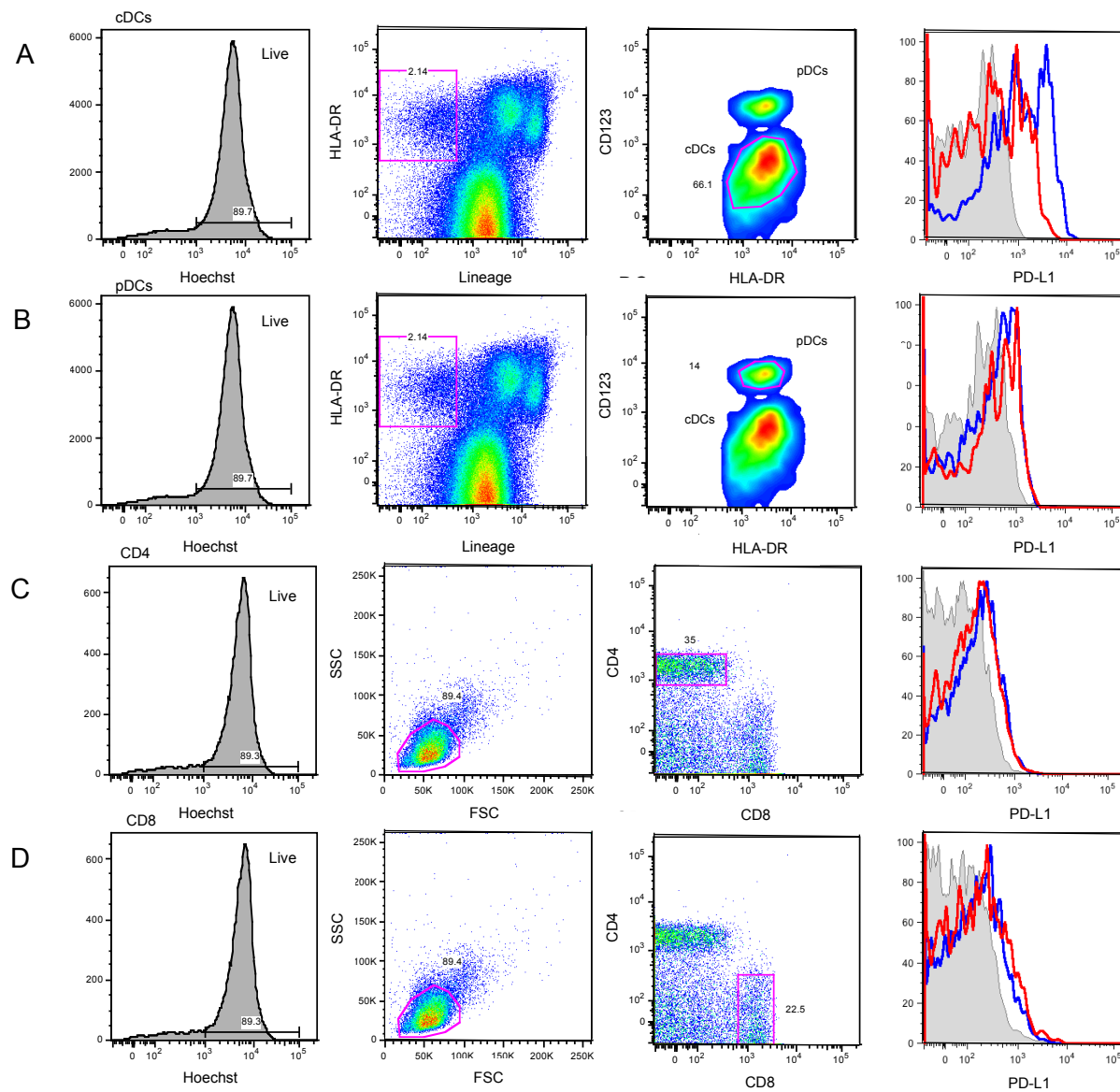

Supplement: Supplementary file 1 — Figure S1. Representative plots of sorted conventional dendritic cells (cDCs) and isolated memory CD4+ T cells (Tm). (a) Gating strategy reflecting the purity of isolated memory CD4+T cells (Tm) characterized by the CD3+CD4+CD45RO+CD45RA− phenotype (purity of 99.6%). (b) Gating strategy for sorting cDCs. (c) Gating strategy to phenotype cDCs, purity of 90.0% of lineage negative cDC population (CD3−, CD14−, CD19−, CD56−), HLA-DR+, and CD123dim. Figure S2. The A(H1N1)pdm09 virus does not induce PD-1 expression;whereas PD-L1 expression on DCs is induced directly by the A(H1N1)pdm09 virus whilst PD-L1 expression on T cells is dependent on the presence of antigen-presenting cells. (a) PBMCs were stimulated with A(H1N1)pdm09 virus (pH1N1), seasonal influenza virus (H3N2), staphylococcal enterotoxin B or synthetic TLR7 agonist (CL264); PD-1 expression in DCs and T cells was analyzed by flow cytometry. Fold increase in PD-1 expression in cDCs and pDCs, CD4+ and CD8+ T cells after 18 h of stimulus. Enriched (HLA-DR+ cell-depleted)T cells and DCs (b) were stimulated with pH1N1, SEB or CL264; PD-L1 expression in DCs and T cells was analyzed by flow cytometry and representative histograms are shown. M: medium. Figure S3. PD-L1 is expressed in cDCs and memory CD4+ T cells after 5 and 7 days of culture with A(H1N1)pdm09. (a) PD-L1 expression on isolated memory CD4+ T cells, 7 days after co-culture with sorted cDCs in the presence (blue) or absence (red) of pH1N1 virus. (b) PD-L1 expression on cDCs cultured for 5 days in the presence (blue) or absence (red) of pH1N1. Figure S4. Gating strategy and representative plots of analyzed dendritic (DCs) and T cells from patients and healthy controls. Gating strategy and representative histograms of PD-L1 expression in cDCs (a, Lin−HLA-DR+CD123dim) and pDCs (b, Lin−HLA-DR+CD123+). Gating strategy and representative histograms of PD-L1 expression in CD4+ T cells (c, CD4+CD8−) and CD8+ T cells (d, CD4−CD8+). The shaded histogram represents P [file 989673.f1.pdf]
